# Effects of food bar chewing duration on the physiologic, metabolic, and perceptual responses to moderate-intensity running

**DOI:** 10.1007/s00421-024-05521-3

**Published:** 2024-06-03

**Authors:** Thomas R. Geaney, Zachary A. Sievert, J. David Branch, Patrick B. Wilson

**Affiliations:** 1 Human Performance Laboratory, School of Exercise Science, College of Health Sciences, Old Dominion University, Norfolk, VA 23529 USA; 2 Department of Rehabilitation, Exercise and Nutrition Sciences, College of Allied Health Sciences, University of Cincinnati, Cincinnati, OH 45221 USA

**Keywords:** Carbohydrate, Exercise, Gut, Metabolism, Sport nutrition

## Abstract

**Purpose:**

Chewing duration can affect food particle size, gastric processing, and postprandial glycemia, but these effects have not been investigated with exercise. This study examined how the chewing duration of a food bar impacts glycemic and metabolic responses, gastrointestinal (GI) symptoms, psychological affect, and performance during endurance running.

**Methods:**

This randomized, unblinded, crossover study had 15 males (35.2 ± 7.4 years, VO_2peak_: 56.1 ± 5.2 ml/kg/min) attend three laboratory visits. Visit 1 required a VO_2peak_ test, 10 min familiarization run at 60% VO_2peak_, and familiarization time-to-exhaustion (TTE) test (10 min at 90% VO_2peak_, followed by TTE at 100% VO_2peak_). Visits 2 and 3 consisted of a 60 min run at 60% VO_2peak_, followed by TTE testing. Participants were fed 45 g of a bar (180 kcal, 4 g fat, 33 g carbohydrate, 3 g protein, 1 g fiber) in 9 g servings 30 min before running, and 27 g of bar in 9 g servings at three timepoints during the 60 min run. Participants consumed the servings in 20 (20CHEW) or 40 (40CHEW) masticatory cycles, at 1 chew/second. Outcomes included blood glucose, substrate use, GI symptoms, perceived exertion (RPE), overall feeling, and TTE.

**Results:**

Post-prandial blood glucose, GI symptoms, and RPE increased over time, but there were no significant between-condition or condition-by-time effects. TTE showed no significant between-condition effect (20CHEW: 288 ± 133 s; 40CHEW: 335 ± 299 s; *p* = 0.240). Overall feeling demonstrated a time-by-condition effect (*p* = 0.006), suggesting possible better maintenance over time with 40CHEW.

**Conclusion:**

Cumulatively, the results suggest that extended chewing minimally impacts physiology, perceptions, and performance during 60 min moderate-intensity running.

## Introduction

Ingesting carbohydrate during endurance exercise can benefit performance by maintaining carbohydrate oxidation and sparing muscle glycogen (Cermak and van Loon 2013). Exogenous carbohydrate feedings are typically consumed as drinks, gels, bars, or a mixture of forms. However, certain sources, such as solid bars, can increase gastrointestinal (GI) discomfort during exercise. Pfeiffer et al. (2010) found that a solid bar led to greater stomach fullness throughout 3 h of moderate-intensity cycling as compared to a drink. While mild stomach fullness may not be a problem for all athletes, especially cyclists, moderate-to-severe stomach fullness has been associated with exercise termination in runners (Daries et al. 2000). Additionally, Guillochon and Rowlands (2017) conducted a crossover experiment in which participants ingested carbohydrate bars, gels, drinks, or a mixture during 140 min of cycling race simulation. The bars, relative to gels and drinks, increased several GI symptoms, and performance was likely worse with bar ingestion relative to gel ingestion.

This potential exacerbation of GI symptoms with ingesting solid bars during exercise may be caused by delayed gastric emptying, as food particles > 3 mm slow transit from the stomach into the small intestine (Boland 2006). Food texture also impacts digestion, with coarser textures delaying gastric emptying, in part, through increased viscosity (Vincent et al. 1995). Alternatively, stomach fullness and other GI symptoms observed with bar ingestion could have been because the bars contained more protein, fat, and total energy than the gels and drinks (Guillochon and Rowlands 2017; Pfeiffer et al. 2010).

Both food particle size and texture can be altered by chewing, which may ultimately have downstream effects on gastric emptying and GI symptoms. Pera et al. (2002), for example, found that 50 chewing cycles of a meal (egg, ham, crackers, water) resulted in smaller food particle size and accelerated gastric emptying as compared to 25 chewing cycles. Mercier and Poitras (1992) recruited 142 women with chewing difficulties and found that 60% of them complained of digestive issues, including burning abdominal pain, bloating, and other GI symptoms. Among those who underwent surgery to improve masticatory function, 85% who initially had abdominal pain reported an improvement after one year. Other studies, however, have found inconsistent results as it relates to the impact of chewing duration and/or efficiency on gastric emptying and GI symptoms (Hattori et al. 2008; Koike et al. 2013; Kumar et al. 2022; Sumonsiri et al. 2019). These equivocal findings could be due to differences in participants studied (healthy vs. masticatory dysfunction), nutrient profile of ingested foods, and methods employed to assess gastric emptying.

Beyond possibly impacting gastric processing, chewing may also influence glycemic responses. In the study by Ranawana et al. (2014), participants chewed spoonfuls of rice 15 times or 30 times. Relative to 15 chews, the glycemic response was 29% higher with 30 chews. Tan et al. (2016) had participants consume 50 g of carbohydrate from rice in 15 min or less and measured mastication parameters using surface electromyography. Overall, there was a positive association between chews per mouthful and glycemic response. Thus, findings from Ranawana et al. (2014) and Tan et al. (2016) imply that more extensive chewing could increase postprandial blood glucose responses.

Despite these findings, there is a lack of research investigating how food chewing time and number of chewing cycles affect physiological and perceptual responses to exercise. Consequently, this study investigated if increasing chewing time/cycles of a solid bar alters glycemic responses, substrate use, GI symptoms, psychological affect, and performance during endurance running. We hypothesized that chewing a bar for 40 cycles, as compared to 20 cycles, would increase blood glucose, increase carbohydrate oxidation, reduce GI symptoms, improve affect, and enhance endurance running performance.

## Methods

### Participants

Recruitment was conducted by distributing flyers locally, word of mouth, and social media posts. Inclusion criteria were that participants had to be: (1) 18–55 years old, (2) running at least 15 miles/week, with at least one 90 min run every two weeks, (3) free of cardiovascular disease, diabetes, pulmonary disease (except controlled asthma), inflammatory bowel disease, swallowing problems, major dental problems, allergies to ingredients in the food bar, Celiac disease, and injuries that interfered with prolonged (i.e., 60–90 min) running.

This study complied with the Declaration of Helsinki. Runners participated voluntarily and signed an informed consent document approved by Old Dominion University’s Institutional Review Board (reference # 22–178) after being briefed on the study’s purpose, procedures, requirements, and potential risks. The study enrolled 20 participants (18 male and 2 females). Of these, 15 males completed all three visits and were included in the analyses. The characteristics of the participants included are shown in Table 1.

**Table 1:** Characteristics of the participants (*n* = 15)

	Mean ± SD or median (25th–75th percentile)
Age (years)	35.2 ± 7.4
Weekly running volume (km)	60.2 ± 24.8
Height (m)	1.80 ± 0.06
Bod pod mass (kg)	77.2 ± 6.9
Bod pod fat (%)	17.4 ± 6.0
GI-symptom history (0–32)	2 (0–5)
VO_2peak_ (mL/kg/min)	56.1 ± 5.2

GI-symptom history scores were derived by asking over the past few months how frequently participants experienced GI symptoms while exercising (0 never, 1 rarely, 2 = sometimes, 3 = frequently, and 4 = almost always). The listed GI symptoms were nausea, reflux/regurgitation, belching, stomach fullness, bloating, abdominal cramps, flatulence, and urge to defecate

A formal sample size calculation was not carried out prior to beginning the study. The target sample size of at least 15 participants was based on a prior crossover experiment showing differences in glycemic responses with altering chewing duration (Ranawana et al. 2014).

### Study design

This study used a 3-visit, randomised, unblinded, crossover design with 2 experimental conditions. In one condition, participants chewed a bar in 40 cycles (40CHEW), at a rate of 1 chew per second. The other condition involved chewing the bar in 20 cycles (20CHEW), at a rate of 1 chew per second. A vanilla chip chewy granola bar (Cascadian Farm Organic, Skagit Valley, WA, USA) was used due to its low fat and protein content. The bar supplied (per 35 g) 140 kcal, 3 g of fat, 26 g of carbohydrate, 2 g of protein, and 1 g of fiber. The bar was fed at specified intervals before and during running (detailed later).

Sex-specific randomisation lists, utilizing block sizes of two and four, were created (using https://www.sealedenvelope.com) by an individual not responsible for data collection. Participants were randomised to 40CHEW or 20CHEW first, then completed the alternative treatment during the next visit. Eight participants received 20CHEW first, while seven received 40CHEW first.

### Testing protocol

Testing occurred at the Human Performance Laboratory at Old Dominion University. The laboratory was temperature-controlled for all visits (20–24 ºC), and participants were instructed to avoid vigorous physical training for 24 h prior to each visit. Caffeine was prohibited for 12 h before arrival. Participants were asked to not ingest any caloric-containing foods or beverages in the 4 h prior to visit 1 (baseline testing), while they reported to the laboratory fasted for at least 8 h on the two experimental test days (visits 2 and 3).

At visit 1, participants completed a questionnaire on demographics, running history, and GI symptom history. Next, height was measured with a stadiometer, while a Bod Pod (COSMED USA, Concord, CA, USA) was used to measure body mass and composition. Participants then performed a VO_2max_ test on a TrackMaster TMX425CP treadmill (Newton, KS, USA). A metabolic cart (TrueOne 2400, Parvo Medics, Salt Lake City, UT, USA) quantified volumes of oxygen consumption and carbon dioxide production (based on 30 s averages of data). The metabolic cart’s gas and flow sensors were calibrated with standard reference gas and a 3 L syringe. Participants wore a Hans Rudolph (Shawnee, KS, USA) facemask connected to a 2-way non-rebreathing valve, which was connected via a hose to the metabolic cart. A chest-strap monitor (Polar H10, Kempele, Finland) was used to monitor heart rate (HR). The treadmill protocol followed Wilson and Ingraham (2015). Briefly, testing began with a 3 min walk at a 5.0 km/h and 0% grade, followed by 1 min stages at 1% grade, with increasing velocity of 0.64 km/h for each stage until the participant reached their self-reported 5 km race speed. Thereafter, the grade increased by 1.5% each minute until volitional fatigue.

Determination of VO_2max_ versus VO_2peak_ was based on evaluating respiratory exchange ratio (RER) and HR relative to an aged-predicted maximum (208—0.7 × age) (Wagner et al. 2020). If a participant achieved an RER ≥ 1.05 and an observed HR of ≥ 90% of the age-predicted maximum, they were deemed to have achieved their VO_2max_. The mean ± SD percentage of age-predicted maximum HR was 98.8 ± 6.7% and the maximum RER was 1.09 ± 0.07. Even though all participants achieved ≥ 90% of their aged-predicted maximum HR, and all but two participants achieved a maximal RER ≥ 1.05, the term VO_2peak_ is used in the remainder of the paper given that VO_2max_ verification testing was not performed.

Next, participants rested for 15 min, which was followed by a 10 min familiarization run at 60% VO_2peak._ This run, and the subsequent experimental trials, were carried out on a T170 DE SPORT MED treadmill (Cosmed, Rome, Italy). An equation from Mayhew (1977) was used to find a treadmill speed equal to 60% of each participant’s VO_2peak_.

Speed (mph) = (relative VO_2_ at 60% of VO_2peak_ / 5.34) + 0.82.

During the familiarization run, VO_2_ was analyzed from 5 to 10 min to confirm that the speed approximated 60% VO_2peak_. Minor speed adjustments were made if the participant’s measured VO_2_ was high or low (e.g., 1 mL/kg/min) relative to what was calculated. Ultimately, the measured VO_2_ during the final min of the familiarization was 34.0 ± 3.1 mL/kg/min, or 60.8 ± 3.1% of VO_2peak_.

Afterwards, participants were familiarized with a time-to-exhaustion (TTE) test of running at 90% VO_2peak_ for 10 min, followed by running at 100% VO_2peak_ until they decided to quit by grabbing the handrails of the treadmill and stepping off the belt. Speeds were derived using the equation from Mayhew (1977) described previously. Within 5 min of completing the familiarization TTE test, participants practiced ingesting two 9 g portions of the bar using 20 and 40 chew cycles, respectively. A 3 portion was offered for additional practice if the participant desired it.

Participants filled out a food log for the 2 days before visit 2 and were asked to, as best they could replicate the same intake before visit 3. Upon arriving for visit 2, participants put on an HR monitor and had baseline measurements taken. Capillary blood glucose was assessed using a FreeStyle Lite monitor (Abbott Diabetes Care Inc., Alameda, CA, USA). Participants rated nine GI symptoms (nausea, belching, regurgitation/reflux, stomach fullness, bloating, side stitching, abdominal cramps, gas, urge to defecate) using a 0–10 questionnaire (Wilson 2017). The Feeling Scale (FS) was used to assess affect (from -5 ‘very bad’ to + 5 ‘very good’) (Hardy and Rejeski 1989).

After baseline assessments, participants ingested 45 g (supplying ~ 33 g of carbohydrate) of a bar 30 min prior to the 60 min run. The bar was served in 5 9 g portions (6.7 g of carbohydrate per portion), and the chewing protocol was either 40CHEW or 20CHEW. The 9 g portion size was selected based on examining reference data on typical bite sizes (Ketel et al. 2019) and through investigator pilot testing. Each subsequent 9 g portion was served with minimal time (< 5 s) from when the prior portion was finished. A metronome (MR-500, Matrix, Korea), along with an investigator counting, was used to cue participants to chew once per second. Participants were offered water ad libitum after finishing each series of bar servings.

Thirty minutes after the first bar serving, participants started the 60 min run at 60% VO_2peak_. This intensity approximates that of many ultra-running races (Schena et al. 2014), the type of event during which solid food is most likely to be consumed. Participants ingested ~ 20 g of carbohydrate (27 g of bar) at 5, 25, and 45 min of the 60 min run, following the same chewing protocol. The bar was served in 9 g portions (6.7 g of carbohydrate) while the participant walked at 3.5 mph. Again, water was offered after feedings, and the amount was recorded with a scale (i500 balance, My Weigh, Phoenix, AZ). Participants drank the same amount of water during the next experimental trial. Median (25th-75th percentile) water intakes were 103 (68–192), 52 (44–87), 57 (42–79), and 61 (41–103) mL at the baseline, 5 min, 25 min, and 45 min feedings, respectively.

**Fig. 1:**
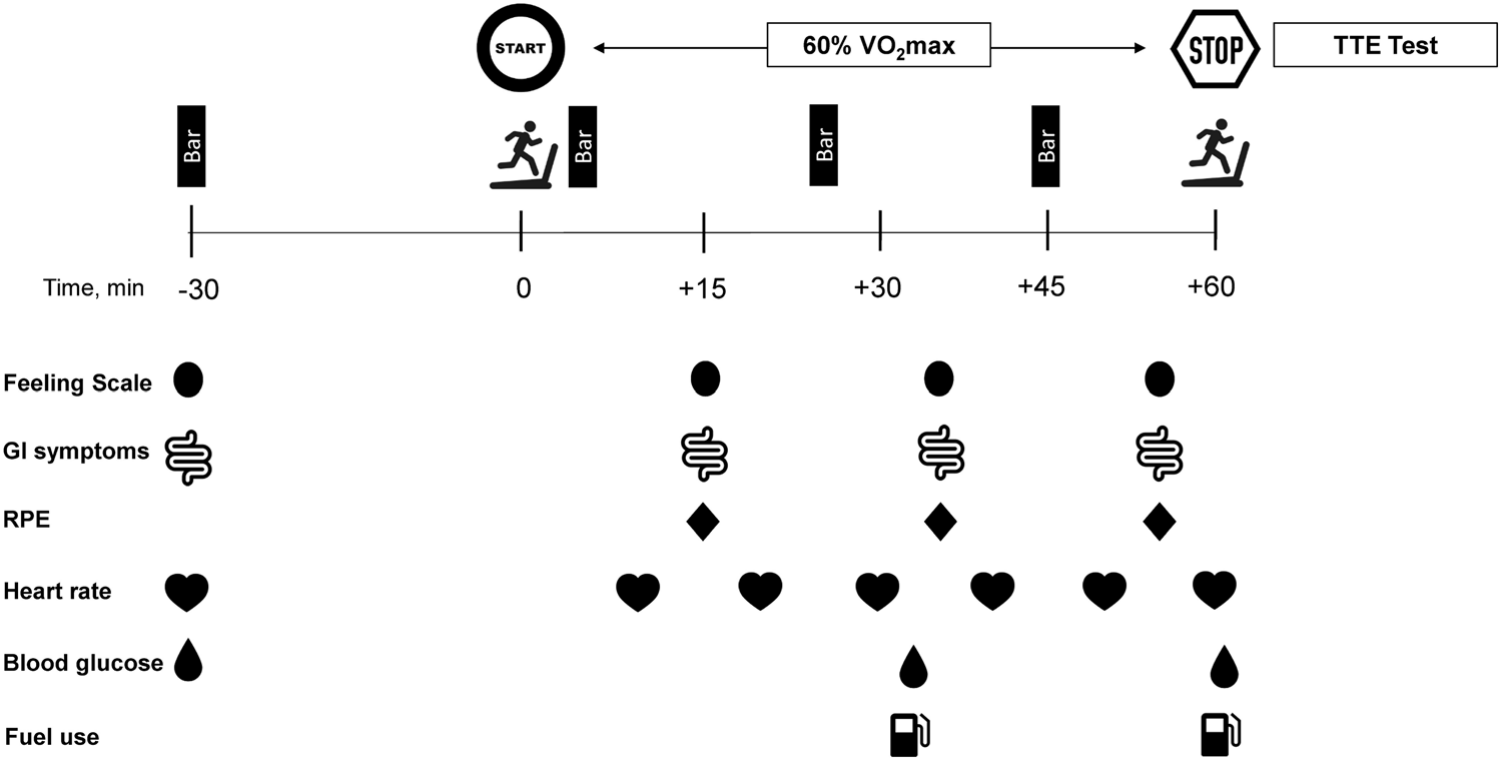
Overview of the study protocol and measurements

The same measurements taken at baseline, along with rating of perceived exertion (RPE; Borg, 1970), were assessed during the 60 min run at various timepoints (see Fig. 1).

Respiratory gases were monitored for 2, 3 min periods, starting at 30 min and 57 min. Carbohydrate and fat oxidation rates were calculated using equations (Jeukendrup and Wallis 2005) based on the last minute of each collection.

Fat oxidation: (1.695 × VO_2_)–(1.701 × VCO_2_).

Carbohydrate oxidation: (4.21 × VCO_2_)–(2.962 × VO_2_).

After the 60 min run, the treadmill was stopped so that blood glucose could be collected and instructions for the TTE test could be reviewed with participants. The treadmill’s distance and time displays were covered so that participants were blinded to performance.

Visit 3 followed the same protocol as visit 2, except participants completed the opposite chewing protocol from visit 2. Note, time spent walking during the 60 min runs varied slightly between the two visits. Participants spent approximately one more minute walking during the 40CHEW condition than the 20CHEW condition.

### Statistical analysis

Given that multiple GI symptoms were assessed across multiple timepoints, sum scores were calculated by adding together individual scores from each timepoint to create total and upper GI (nausea, belching, regurgitation/reflux, fullness) symptom variables.

Inspection of histograms and Shapiro–Wilk tests were used to evaluate if the data were normally distributed. The FS, RPE, blood glucose, HR, and substrate use were compared between trials using 2-way ANOVAs with condition (20CHEW vs. 40CHEW) and time (FS, GI: baseline, + 15, + 35, + 55 min; blood glucose: baseline, + 33, post-run; HR: baseline, + 9, + 19, + 29, + 39, + 49, + 59 min; carbohydrate and fat use: + 33, + 60 min) as within-subjects factors. Greenhouse–Geisser p-values are reported in cases where the assumption of sphericity was violated. In the case of time effects with no interaction effect, pairwise comparisons with a Bonferroni correction were applied. In the case of interaction effects, between-condition effects at each timepoint were explored using paired *t*-tests with Bonferroni adjustments for multiple comparisons. TTE performance was compared using a paired *t*-test. GI symptom variables were non-normally distributed; thus, the Wilcoxon signed-rank test was used. Change scores from baseline to + 55 min were calculated and used to reduce the number of statistical tests and risks of multiplicity.

SPSS Statistics 29 (IBM, Armonk, NY, USA) was used for the analyses. Unless otherwise indicated, normally distributed summary continuous data are reported as mean ± SD, and skewed data are reported as median (25th–75th percentile). Hedges’ *g* was calculated as an indicator of effect size, with the average of variances used in the denominator. Effects of 0.2, 0.5, and 0.8 were deemed small, moderate, and large, respectively. The alpha level was set at 0.05.

## Results

### Physiological data

**Fig. 2:**
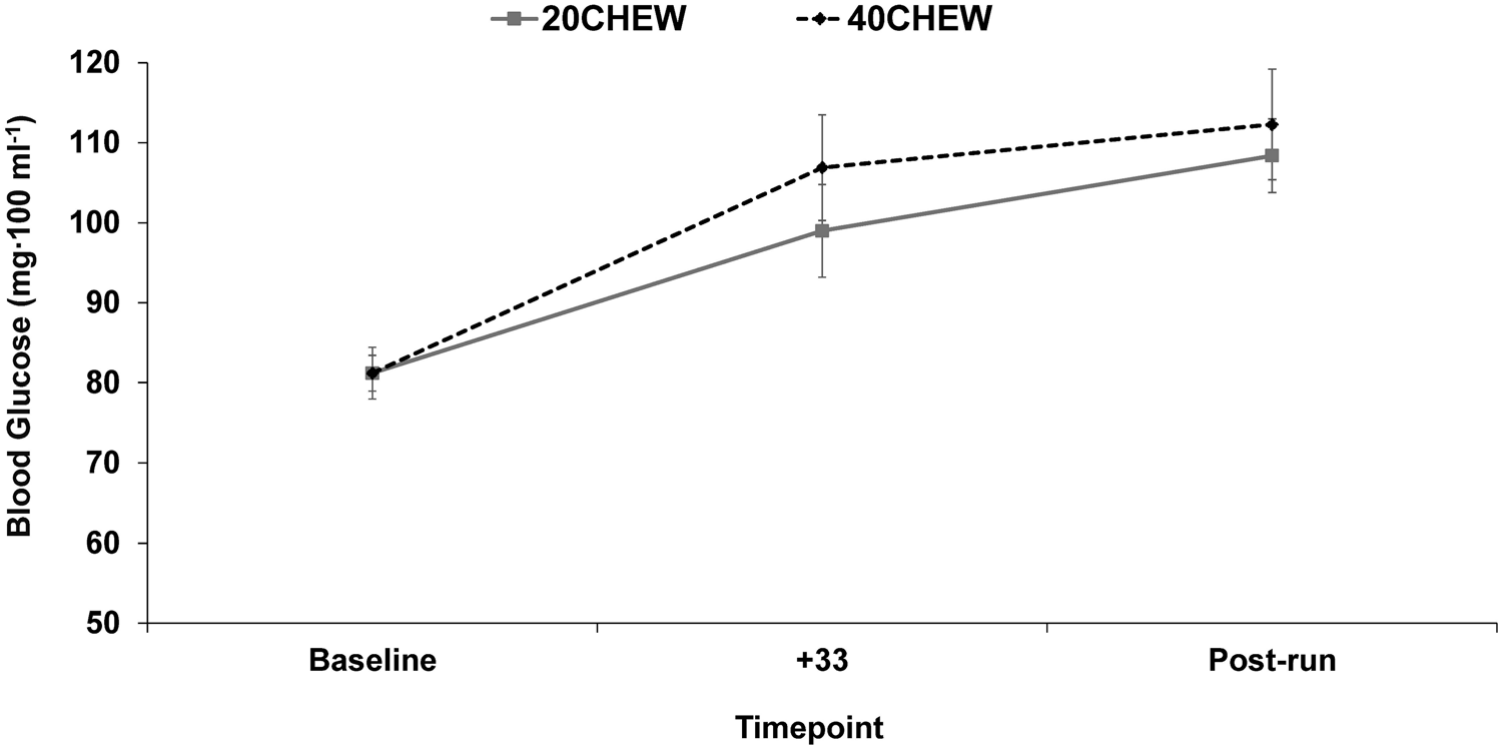
Effect of chew duration on blood glucose before, during, and after treadmill exercise. Values are mean ± SE

Figure 2 shows blood glucose data. There was a significant increase over time (*F* = 16.02, *p* < 0.001), but no significant differences between conditions (*F* = 1.06, *p* = 0.321), nor was there a significant time-by-condition effect (*F* = 1.32, *p* = 0.282). Post hoc testing revealed that blood glucose at + 33 min and post-run were higher (*p* < 0.05) than baseline. Between-condition Hedges’ *g* values at + 33 min (−0.31, 95% confidence interval [CI], −0.80 to 0.19) and post-run (−0.16, 95% CI: −0.64 to 0.32) indicate small effects of lower blood glucose with 20CHEW.

Table 2 displays substrate use data. There was no significant time (*F* = 2.19, *p* = 0.161), condition (*F* = 0.05, *p* = 0.832), or time-by-condition effects (*F* = 0.78, *p* = 0.393) for carbohydrate oxidation. Likewise, there were no significant time (*F* = 1.87, *p* = 0.193), condition (*F* = 0.181, *p* = 0.677), or time-by-condition effects (*F* = 1.88, *p* = 0.192) for fat oxidation.

**Table 2:** Substrate use at minutes 33 and 60 of the 60 min run

	Carbohydrate oxidation (g/min)	Fat oxidation (g/min)
20CHEW		
33 min	1.76 ± 0.35	0.60 ± 0.20
60 min	1.80 ± 0.29	0.60 ± 0.17
40CHEW		
33 min	1.72 ± 0.33	0.62 ± 0.23
60 min	1.87 ± 0.35	0.56 ± 0.21

HR data are shown in Table 3. There was an increase in HR with time (*F* = 476.45, *p* < 0.001) as expected, but there were no condition (*F* = 1.67, *p* = 0.218) or time-by-condition (*F* = 0.41, *p* = 0.646) effects. Based on post hoc testing, baseline and + 9 min were lower than all other timepoints (*p* ≤ 0.001), + 39 min was higher than + 19 min and + 49 min (*p* < 0.05), and + 59 min was higher than + 29 min (*p* < 0.05).

**Table 3:** Heart rate at baseline and during the 60 min run at 60%VO_2peak_

	Baseline	+ 9 min	+ 19 min	+ 29 min	+ 39 min	+ 49 min	+ 59 min
20CHEW	64 ± 14	139 ± 11	143 ± 13	143 ± 11	146 ± 12	144 ± 12	147 ± 12
40CHEW	62 ± 11	137 ± 11	144 ± 15	142 ± 13	145 ± 16	142 ± 14	145 ± 14

### Perceptual data

In 20CHEW, total GI symptoms increased by a median of 5.0 (2.0–9.0) from baseline to + 55 min. In 40CHEW, total GI symptoms increased by 5.0 (1.0–8.0), and there was no difference in change scores between conditions (*Z* = −0.09, *p* = 0.929). Upper GI-symptom scores increased by a median of 3.0 (1.0–5.0) and 4.0 (2.0–6.0) in 20CHEW and 40CHEW, respectively. There was no significant difference in change scores (*Z* = −0.44, *p* = 0.661).

**Fig. 3:**
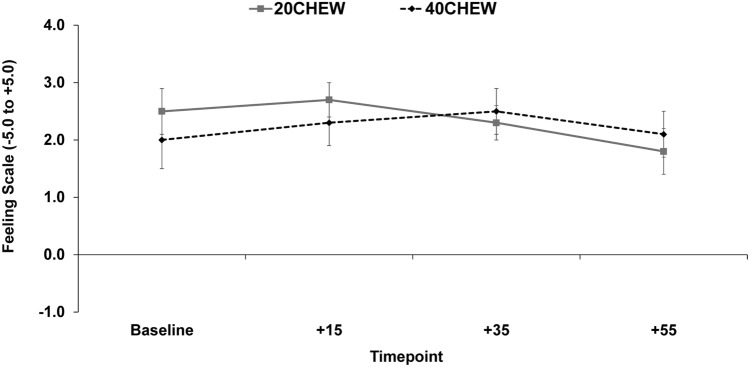
Effect of chew duration on the Feeling Scale before, during, and after treadmill exercise. Values are mean ± SE

For FS data (Fig. 3), there was no time effect (*F* = 1.24, *p* = 0.298) or condition effect (*F* = 0.06, *p* = 0.810), but there was a time-by-condition effect (*F* = 4.78, *p* = 0.006). In other words, FS was relatively stable at 40CHEW but declined with 20CHEW. However, pairwise testing revealed no significant differences between conditions at any individual timepoint.

Values for RPE at + 15, + 35, and + 55 min were 9.5 ± 2.0, 10.5 ± 1.4, and 10.9 ± 1.2 with 20CHEW, while corresponding values with 40CHEW were 9.2 ± 1.8, 9.6 ± 1.8, and 10.0 ± 1.8. There were no condition (*F* = 3.26, *p* = 0.093) or time-by-condition (*F* = 2.74, *p* = 0.082) effects, but RPE did increase over the 60-min run (*F* = 13.66, *p* = 0.001). Pairwise post hoc testing showed that + 35 and + 55 min were higher than + 15 min, and that + 55 min was higher than + 35 min (all *p* < 0.05).

### Performance

**Fig. 4:**
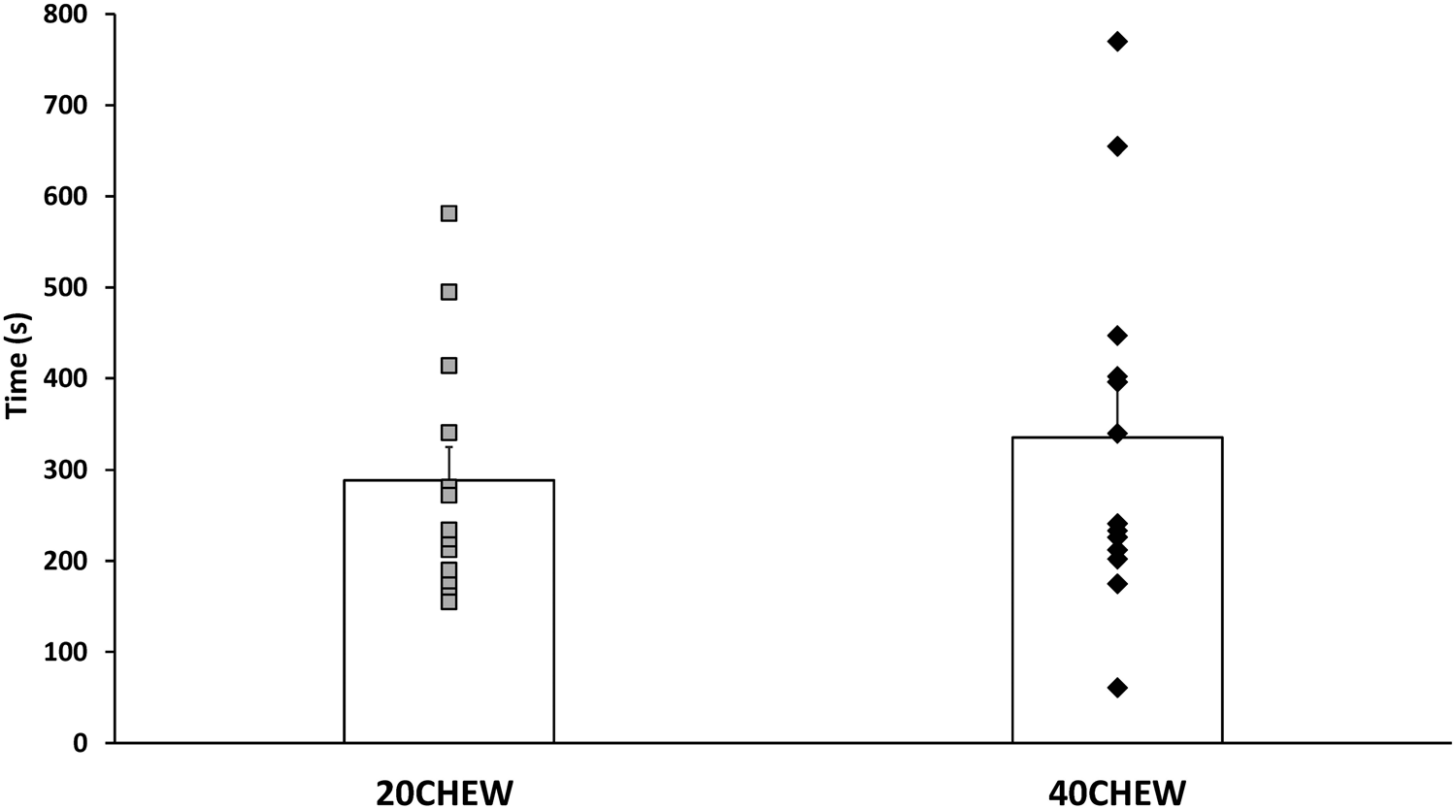
Effect of chew duration on the time-to-exhaustion performance. Values are mean ± SE

Two participants elected not to complete TTE testing (1 due to concern over a tight hamstring, and 1 due to worries it would impact his training). Participants lasted 288 ± 133 s with 20CHEW and 335 ± 199 s with 40CHEW (Fig. 4). The paired differences from the *t*-test showed a non-significant mean difference of −47 ± 137 s (*t*-value = −1.24, *p* = 0.240). A Hedges’ *g* indicates the effect size was small −0.26 (95% CI:−0.77 to 0.26).

## Discussion

This study investigated how chewing impacts glycemic responses, substrate use, GI discomfort, perceived effort/feeling, and performance during 60 min of moderate-intensity running. We hypothesized that there would be higher blood glucose, greater carbohydrate oxidation, less severe GI symptom changes, greater psychological well-being, and longer TTE in 40CHEW than 20CHEW. With respect to blood glucose, there was a significant time effect but no condition or time-by-condition effects. In line with other studies that have fed carbohydrate-rich bars before and during exercise (Horowitz et al. 1999; Mason et al. 1993), our results confirm that blood glucose increases as food is ingested. However, the number of chewing cycles is likely not impactful on blood glucose responses during one hour of exercise at a moderate intensity. It is possible that our conditions did not create a large enough contrast in bolus composition to elicit a difference in blood glucose. Another possibility is that any differences in blood glucose were minimized with the onset and continuation of exercise (Kirwan et al. 2001). That said, a true small effect of 40CHEW on blood glucose cannot be ruled out due to our modest sample size (the Hedges’ *g* for blood glucose at + 33 min was −0.31). Regardless, the practical importance of such a small effect is unclear. A recent observational study of ultra-runners partaking in a 100-mile race found that faster finishers seemingly better maintained steady glucose levels throughout the race as compared to slower finishers, which the authors attributed to higher carbohydrate intakes during the first half of the race (Inamura et al. 2024). At a minimum, this suggests that modest differences in blood glucose homeostasis could alter performance during ultra-endurance running.

There were also no significant differences in carbohydrate or fat oxidation. Given the lack of differences in glycemic responses between 40 and 20CHEW, it is perhaps not surprising that there were no effects on total substrate use. Furthermore, although carbohydrate ingestion during exercise has been clearly shown to lead to better blood glucose maintenance and carbohydrate oxidation versus a placebo, the effects typically do not become large until at least 90 min into exercise (Coggan and Coyle 1991). Both of our conditions supplied the same amount of carbohydrate, and it appears that manipulating chewing duration does not lead to substantial enough differences in GI processing to alter fuel use during 1 h of exercise. In addition, this study did not evaluate exogenous carbohydrate oxidation specifically, which would have provided valuable insight into whether chewing duration influences carbohydrate utilization from endogenous versus exogenous sources. Research that has looked at exogenous carbohydrate oxidation of bars has generally found small differences with drinks and gels (Pfeiffer et al. 2010).

Regarding GI symptoms, there was no effect on chewing duration. One possibility is that the contrast in chewing durations was not enough to result in meaningful downstream digestive effects. Non-exercise research findings are equivocal. Mercier and Poitras (1992) noted that improved masticatory function post-surgery resulted in reduced digestive symptoms. In addition, chewing a meal for 50 cycles resulted in smaller average food particle size and faster gastric emptying as compared to 25 chewing cycles in Pera et al. (2002). In contrast, Poitras et al. (1995) investigated the effects of mastication on gastric emptying with and without a dental prosthesis and showed no differences between conditions. Inducing a larger difference in mastication cycles (i.e., 20 vs. 50–60 chews) could have increased the probability of obtaining meaningful effects in the present study, but that degree of extensive chewing is probably not realistic in an endurance running race, where a goal of many competitors, particularly elite runners, is to avoid slowing down for extensive periods of time to fuel. However, the benefit of moving through aid stations fast and chewing quickly is probably more important in shorter ultra-races than in longer races (Martínez-Navarro et al. 2021).

Other explanations for the lack of GI-symptom differences include the relatively short duration and intensity of exercise. Gaskell et al. (2023), for example, showed that GI symptoms were ranked highest when participants performed 2–3 h treadmill runs at 60% VO_2max_, compared to 1 h of running at the same intensity. Other research has reported that higher exercise intensities are associated with more GI disturbances (Edwards et al. 2021; Wilson 2018). At a duration of 1 hour and an intensity of 60% VO_2peak_, our protocol likely acted as a mild stressor to the GI system. Future research might consider employing more challenging protocols, either by extending the exercise duration, varying the intensity, or by exposing participants to heat.

The difference in TTE between conditions showed a small, non-significant effect size (Hedges’ *g* = −0.26, *p* = 0.240). The lack of benefit is likely related to the fact that there were no differences in blood glucose, substrate use, and GI symptoms, as these are known modifiers of performance. For example, Rowlands et al. (2012) compared ingesting composite versus transportable carbohydrate solutions on cycling performance. The single transportable carbohydrate solution reduced performance by increasing GI discomfort. Likewise, Guillochon and Rowlands (2017) noted that GI symptoms were higher and performance was reduced while ingesting solid bars, in comparison to ingesting carbohydrate drinks and gels. Cumulatively, this body of evidence shows that feeding protocols that inadvertently induce GI symptoms can negatively impact performance. Our data do not support any negative effects of a shorter chewing duration on GI symptoms or performance, but additional studies should attempt to verify this with exercise protocols that simulate the duration of ultra-running.

There were no significant time or condition effects in FS scores, but there was a significant time-by-condition effect. This suggests that the degree of chewing might impact overall feeling while exercising. Reasons for the time-by-condition effect for FS are uncertain, especially given the lack of differences in other outcomes. It is possible that the effect is due to our inability to fully control for factors like stress, sleep, and outside training.

There are certain strengths and limitations to this study. Chewing rate was tightly controlled with a metronome and investigator verbal cues, and the exercise intensity (60%VO_2peak_) and carbohydrate delivery rate (~ 60 g/h during exercise) were chosen for optimization of external validity to ultra-running. In terms of limitations, outside factors such as sleep and stress were not measured or tightly controlled, and our sample of 15 participants is insufficient to detect small effects. Further, a longer-running protocol would have likely led to greater blood glucose and substrate use changes, which could have been helpful for eliciting differences between treatments. Furthermore, insulin responses and exogenous carbohydrate oxidation were not measured, both of which would have been informative. Lastly, there was no pure control trial (i.e., no feeding), which prevents us from deciphering whether there are any benefits or harms of fueling with the bars (irrespective of chewing duration).

## Conclusions

This study’s results indicate that chewing duration has little impact on glycemic responses, overall substrate use, and perceptual responses to 1 h of moderate-intensity running, as well as on subsequent TTE performance. Consequently, runners partaking in ultra-endurance events likely do not need to concern themselves with how thoroughly they chew solid foods during competition. Future studies examining the impact of chewing on glycemic responses, GI discomfort, substrate use, and performance should consider extending treadmill running to a longer duration, increasing the exercise intensity, and/or imposing other GI stressors like heat. Measuring other variables such as insulin and exogenous carbohydrate oxidation would also help to clarify the impact of chewing on the responses to exercise.

## Data Availability

The datasets generated during and/or analysed during the current study are available from the corresponding author upon reasonable request.

## Abbreviations

20CHEW: 20 Chews at a rate of 1 chew per second
40CHEW: 40 Chews at a rate of 1 chew per second
ANOVA: Analysis of variance
CI: Confidence interval
FS: Feeling scale
GI: Gastrointestinal
HR: Heart rate
RER: Respiratory exchange ratio
RPE: Rating of perceived exertion
SD: Standard deviation
TTE: Time-to-exhaustion
VO_2_: Volume of oxygen consumption
VO_2peak_: Peak oxygen consumption during the maximal graded exercise test
